# Integration of publicly available case-based data for real-time coronavirus disease 2019 risk assessment, Japan

**DOI:** 10.5365/wpsar.2022.13.1.889

**Published:** 2022-03-31

**Authors:** Kota Ninomiya, Mariko Kanamori, Naomi Ikeda, Kazuaki Jindai, Yura K Ko, Kanako Otani, Yuki Furuse, Hiroki Akaba, Reiko Miyahara, Mayuko Saito, Motoi Suzuki, Hitoshi Oshitani

**Affiliations:** a Data Management Team, The National COVID-19 Cluster Taskforce, The Ministry of Health, Labour and Welfare, Tokyo, Japan.; b Graduate School of Pharmaceutical Sciences, The University of Tokyo, Tokyo, Japan.; c National Institute of Public Health, Saitama, Japan.; d Jumonji University, Saitama, Japan.; e Department of Virology, Tohoku University Graduate School of Medicine, Sendai, Japan.; f Department of Healthcare Epidemiology, Kyoto University, Kyoto, Japan.; g Center for Surveillance, Immunization and Epidemiologic Research, National Institute of Infectious Diseases, Tokyo, Japan.; h Institute for Frontier Life and Medical Sciences, Kyoto University, Kyoto, Japan.; i Hakubi Center for Advanced Research, Kyoto University, Kyoto, Japan.; * These authors contributed equally.

## Abstract

In response to the outbreak of coronavirus disease 2019 (COVID-19) in Japan, a national COVID-19 cluster taskforce (comprising governmental and nongovernmental experts) was established to support the country’s Ministry of Health, Labour and Welfare in conducting daily risk assessment. The assessment was carried out using established infectious disease surveillance systems; however, in the initial stages of the pandemic these were not sufficient for real-time risk assessment owing to limited accessibility, delay in data entry and inadequate case information. Also, local governments were publishing anonymized data on confirmed COVID-19 cases on their official web sites as daily press releases. We developed a unique database for nationwide real-time risk assessment that included these case lists from local government web sites and integrated all case data into a standardized format. The database was updated daily and checked systematically to ensure comprehensiveness and quality. Between 15 January 2020 and 15 June 2021, 776 459 cases were logged in the database, allowing for analysis of real-time risk from the pandemic. This semi-automated database was used in daily risk assessments, and to evaluate and update control measures to prevent community transmission of COVID-19 in Japan. The data were reported almost every week to the Japanese Government Advisory Panel on COVID-19 for public health responses.

The establishment of reliable and real-time epidemiological data on emerging and re-emerging infectious diseases is crucial for understanding transmission patterns and assessing the impact of public health intervention to mitigate outbreaks. (*1*) However, during public health emergencies, routine surveillance channels can be overwhelmed; thus, real-time assessment may be hampered by insufficient information. (*2*)

In 1981, the Government of Japan established a laboratory-based surveillance system for infectious diseases. In 1999, this was expanded to include a system for patient reporting, and the Act on the Prevention of Infectious Diseases and Medical Care for Patients with Infectious Diseases (hereafter, the Infectious Diseases Control Law) was enacted. (*3*) Collected data were integrated into this national surveillance system: the National Epidemiological Surveillance of Infectious Diseases (NESID) programme. (*3*) To respond to emerging or re-emerging diseases not defined in the surveillance system, surveillance of “undiagnosed serious infectious illness” is included in NESID to promote early detection of pathogens during public health emergencies. (*4*) This surveillance system was used to detect and monitor coronavirus disease 2019 (COVID-19) cases (*5*) even before COVID-19 was labelled as a “designated infectious disease” in Japan by cabinet order on 28 January 2020. (*6*) The designation mandated that health-care providers report all confirmed cases to public health centres in their local jurisdictions under the Infectious Diseases Control Law. (*3*) Thereafter, the collected data were confirmed by local governments and ultimately reported to the Ministry of Health, Labour and Welfare (MHLW).

The government also established the national COVID-19 cluster taskforce (comprising governmental and nongovernmental experts) on 25 February 2020, to support the MHLW’s efforts. (*7*) The taskforce members analysed current case and cluster data, conducted daily risk assessments and provided technical advice for public health decision-making. The surveillance data from NESID were initially used for this analysis; however, the data were not available to nongovernmental experts owing to privacy issues. Also, the surveillance data were inadequate for timely analyses of community transmission of this emerging infection because they were not designed to report cases on a day-to-day basis. The standard surveillance process involves health-care facilities that diagnose cases sending case information to the public health centre in their jurisdiction via fax for registration; at the health centre, the data are manually entered into the system for further verification and reporting. (*3*) As the COVID-19 case numbers grew, the reporting process became overwhelmed, leading to significant delays in reporting to public health authorities. Also, the standard reporting form did not include variables essential for risk analysis of COVID-19 transmission, such as history of contact with confirmed cases, being in a possible spreading event or travel history from an epidemic area or country. To alleviate these issues, the MHLW asked local governments to send daily case information in a spreadsheet directly to the MHLW via e-mail. (*8*)

This updated surveillance process also had reporting delays and discrepancies between the number of cases reported by local governments and those reported by the MHLW as the pandemic unfolded throughout Japan. The reporting delay by local governments to the MHLW could exceed 1 week in some instances. Data entry errors such as spelling and typographical errors were also recognized. These shortcomings made it difficult for the national COVID-19 cluster taskforce to analyse the epidemiological situation and conduct real-time risk assessment to inform decision-making.

Therefore, another system of data collection was urgently needed to collect and integrate case information for more accurate situation analysis and rapid risk assessment to bolster public health decision-making. In this report, we describe the development of such a database that supported real-time risk assessment and its impact on policies for managing the COVID-19 pandemic.

## Database development

The new database used publicly available data from daily press releases published on local government web sites. (*9*) Each local government releases anonymized individual case data and aggregated daily case numbers on their web site. Although the Cabinet Secretariat (https://cio.go.jp/policy-opendata) recommended that local governments share data with the public in a universal format (e.g. a file of comma-separated values) in line with the five-star open data model, (*10*) the press release format was not standardized or consistent across local governments, especially during the early phase of the pandemic. Some press releases were published as PDF files or embedded directly in the HTML, whereas others described cases in text not in tables. Therefore, the case information needed to be converted and integrated into a standardized data format within a single database.

Initially, we extracted the case information manually from each local government web site with support from volunteers; however, this was labour-intensive and required significant resources. Subsequently, we developed a programme written in Python programming language (*11*) to automate extraction of information directly from the web sites and its conversion to tabular form.

The database contained the following variables for each case: official reporting date, reporting prefecture, prefectural case identification number, reporting municipality, municipal case identification number, age, sex, occupation, residence (limited to prefecture and municipality), onset date, confirmation date, presence of symptoms at the time of confirmation, history of overseas travel, history of domestic travel and epidemiological link with other confirmed cases.

## Database management

**Fig. 1:**
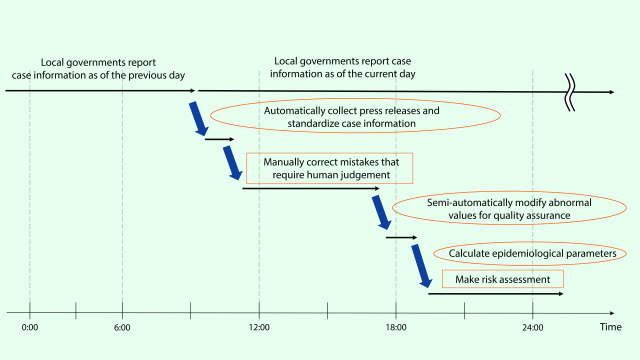
Daily operating procedures of the COVID-19 database, Japan

Initially, quality assurance of the data was performed manually, with an algorithm developed in Python to detect possible data errors, including abnormal values, missing data, inconsistencies in Japanese characters (Kanji) and categorical variables (e.g. occupation and place of residence). These errors were corrected daily. We developed a standard operating procedure for updating case information without errors (**Fig. 1**). The database automatically collected press releases from web sites in the morning, then a manual verification process was used to ensure the data entry was correct. Next, we semi-automatically modified abnormal values before calculating epidemiological parameters, which were then used by the cluster taskforce for their risk assessment. Close collaboration with data users, such as governmental officers and experts, allowed their feedback to improve database development and maintenance.

[**insert** Figure 1]

## Results

The database included 776 459 cases from 15 January 2020 to 15 June 2021 (**Table 1**), from which daily epidemic curves and geographical maps were created. (*12*) Epidemiological information on the first few hundred cases was derived from the database. (*13*) Further epidemiological parameters (e.g. effective reproduction number, proportion of unlinked cases, age and sex distribution, symptom onset and confirmation delay) were derived from the database for real-time risk assessment. An analysis of clusters in the early phase showed that most cases were younger and either pre-symptomatic or asymptomatic. (*14*)

**Table 1:** List of variables included in the COVID-19 database from 15 January 2020 through 15 June 2021 (*n* = 776 459), Japan

Variables	Description	Coverage, *n*(%)
Reporting prefecture	Name of the prefecture where the case was reported	776 459 (100)
Case identification number by prefecture	Serial number of the case in the reporting prefecture	720 726 (92.8)
Reporting municipality	Name of the municipality where the case was reported	275 149 (35.4)
Case ID by municipality	Serial number of the case in the reporting municipality	263 564 (33.9)
Age	Age stratum by decade	668 250 (86.1)
Sex	Male or female	662 444 (85.3)
Occupation	Case’s occupation type: health-care professional, public servant, office worker, corporate executive, educational professional, self-employment, unemployment, other	386 874 (49.8)
Residential prefecture	Name of the prefecture where the case resides	692 084 (89.1)
Residential municipality	Name of the municipality where the case resides	506 075 (65.2)
Onset date	Date of illness onset	402 855 (51.9)^a^
Date of confirmation	Date that SARS-CoV-2 infection was confirmed	540 027 (69.5)
Date of official announcement	Date the case was announced by the prefecture or municipality	776 024 (99.9)
Presence of symptoms at the time of confirmation	Whether the case had any symptoms when SARS-CoV-2 infection was confirmed	416 837 (53.7)
History of overseas travel	Whether the case had a history of overseas travel before the infection was confirmed	474 (0.1)
History of domestic travel	Whether the case had a history of domestic travel before the infection was confirmed	13 558 (1.7)
Epidemiological link	Whether the case had a history of close contact with other positive cases or a history of staying in a clustered location known to be associated with more than one infected case before the infection was confirmed	326 693 (42.1)
Re-positive	If an infected person tested positive again after testing negative	353 (0.05)

^a^ These calculations included asymptomatic cases. If asymptomatic cases were excluded (**N** = 710 553), the coverage of onset date would be 56.3% (*n* = 400 350).

Epidemiological analysis and real-time risk assessment results based on this database have been presented to the Japanese Government Advisory Panel on COVID-19 to evaluate the effectiveness of public health measures against COVID-19. The government’s COVID-19 dashboard (https://covid19.mhlw.go.jp), which uses data not accessible to the public, was also refined to be used for the visualization of epidemiological data in parallel with our database. Because our database and the government’s database were not cross-checked or merged, the Advisory Panel analysed data summaries from both databases to identify discrepancies between them and improve their quality. Furthermore, comparing analyses from both data sets provided a multifaceted perspective to help experts conduct risk assessments.

## Discussion

The development of a reliable database during a public health emergency is challenging. High numbers of cases can overwhelm pre-existing surveillance systems, necessitating an alternative system. Basic demographic information derived from a reliable database is required for real-time risk assessment and policy-making. We successfully developed an integrated database platform that collected press release information from local government web sites. Similar processes have been used in other Asian countries and regions, such as China, Taiwan (China), Hong Kong Special Administrative Region (China) and Singapore, where data from government-issued press releases on new COVID-19 cases have been used for studies to investigate transmission patterns or to evaluate interventions. (*15*, *16*) Our database was used for conducting research activities, and also served as a real-time monitoring tool to support public health decision-making in the outbreak setting.

Balancing privacy protection with the need for granular personal data for public health analyses during health emergencies has been controversial. Using press release data that do not include identifying information (as per local government policy) allowed for the sharing of outputs with nongovernmental officers with minimal risk to private information. The database provided consistent reporting of cases nationally, which in turn allowed for information to be shared among local governments, particularly where cases had travelled to multiple prefectures.

One of the limitations of this database is that the reporting format for some variables differed across local governments and phases. Also, some variables were partially or entirely missing from the data recorded by some local governments, (*9*) and local reporting forms were sometimes reformatted without notification, requiring updating of the database processes; hence, it was difficult to share consistent coding scripts for collecting information. Furthermore, the quality of the data from each local government varied depending on workload, especially for data on epidemiological links from active surveillance at public health centres. Detailed clinical data and disease outcomes were not publicly available.

Despite the limitations, this semi-automated database based on publicly available data was useful for monitoring pandemic trends, conducting real-time risk assessment and assessing the impacts of public health policies. No additional cost for computing resources or software was required apart from a few personal computers to manage our database; also, abstracting sufficient data from open-source data was technically straightforward. Therefore, our method could be adapted for use in resource-constrained settings and could serve as a meaningful model for other countries to create similar databases during public health emergencies. Based on this experience, we recommend that countries develop legislation and establish systems that can extract and store anonymized case information from publicly provided information to supplement routine surveillance systems.
